# Microbiological Findings and Clinical Outcomes in Ugandan Patients with Infected Burn Wounds

**DOI:** 10.3390/ebj4010007

**Published:** 2023-02-07

**Authors:** Johannes Weinreich, Christina Namatovu, Sara Nsibirwa, Leah Mbabazi, Henry Kajumbula, Nadine Dietze, Christoph Lübbert, Hawah Nabajja, Joseph Musaazi, Charles Kabugo, Amrei von Braun

**Affiliations:** 1 Division of Infectious Diseases and Tropical Medicine, Department of Medicine II, Leipzig University Medical Center, Liebigstrasse 20, 04103 Leipzig, Germany; 2 Burns Unit, Kiruddu National Referral Hospital, Salaama Road, Makindye District, Kampala P.O. Box 2 4092, Uganda; 3 Infectious Diseases Institute, College of Health Sciences, Makerere University, Kampala P.O. Box 7072, Uganda; 4 Department of Microbiology, Makerere University, Kampala P.O. Box 7072, Uganda; 5 Institute of Medical Microbiology and Virology, Leipzig University Medical Center, Johannisallee 30, 04103 Leipzig, Germany

**Keywords:** nosocomial wound infections, burns, Gram-negative bacteria, antimicrobial resistance, Uganda

## Abstract

Nosocomial wound infections are a dreaded complication in patients with burns. However, access to the necessary microbiological diagnostics is impaired in low-resource settings. This prospective observational cohort study aimed to describe the bacterial pathogens, resistance profiles and clinical outcomes of patients with wound infections admitted to the largest specialized unit for burns and plastic surgery in Uganda. Blood and wound swab cultures were taken for bacterial species identification and antibiotic susceptibility testing. A total of 140 patients (female: *n* = 62, 44.3%) with a median age of 26 (IQR 7–35) years were included between October 2020 and April 2022, of which the majority (*n* = 101, 72.2%) had burn wounds (72.3% Grade 2b, 14.9% Grade 3). Gram-negative *Enterobacterales*, *Pseudomonas* spp. and *Acinetobacter* spp. were most commonly isolated from wound swabs and nearly all isolates were multidrug resistant with very limited treatment options. While the clinical outcome was favorable in 21 (15%) study participants, the majority were left with disabilities (minor: *n* = 41, 29.3%, moderate: *n* = 52, 37%, major: *n* = 14 (10%)). Twelve (8.6%) study participants died, mostly of Gram-negative sepsis. Our findings highlight the urgent need for routine access to microbiological diagnostics to improve patient care and local surveillance efforts on antimicrobial resistance.

## 1. Introduction

Severe bacterial wound infections are common complications in trauma and burn patients [1]. A low socioeconomic background, lack of preventative measures, as well as impaired access to primary healthcare facilities are important risk factors for high infection rates in burn wounds, especially in low and middle income countries (LMIC) [2,3,4]. Additionally, colonization with multidrug resistant pathogens and nosocomial infection are frequently found [5]. Patients requiring skin grafts and plastic surgical reconstruction suffer from infections due to resistant pathogens disproportionately [6]. Therefore, the demand for antimicrobial stewardship (AMS) interventions, local surveillance data and regularly updated empirical treatment guidelines is high [7].

Globally, antimicrobial resistance (AMR) is on the rise, accounting for a continuously increasing number of deaths due to virtually untreatable infections [8,9,10]. In human health, the inappropriate choice of antibiotic drugs, inadequate dosing, poor adherence to treatment and antimicrobials of poor quality foster the development and spread of AMR [11]. Inadequate measures to control the spread of infections with resistant bacteria inside and outside of medical facilities are an additional factor.

However, resource-limited settings such as public healthcare facilities in sub-Saharan Africa often lack the financial resources for routine microbiological diagnostics in clinical care and for surveillance. Thus, too little is known about the magnitude and dynamics of AMR in these regions [12,13]. In addition to the lack of surveillance, high barriers for the implementation of AMS programs, and an overall increase in antibiotic consumption lead to an increasing burden of AMR in LMIC [14,15,16]. For instance, a recent observational study on AMR in bloodstream infections among Ugandan patients found that a substantial proportion of pathogens were resistant to first-line antibiotic drugs (e.g., fluoroquinolones, penicillin, ceftriaxone) at much higher rates than those reported from high-income countries [17]. Results from studies on wound infections, as well as on potential environmental sources of resistant bacteria also suggest a high burden of AMR in the region [18,19]. These and other retrospective studies are, however, limited by a small sample size, likely due to patient-incurred costs for diagnostics and the limited availability of microbiology supplies in routine care.

To the best of our knowledge, this is the first prospective study on the bacterial pathogens and clinical outcomes of Ugandan patients with wound infections following burns and other wounds requiring skin grafting. The overall objective of this study was to report accurate clinical and microbiological data from patients with wound infections recruited at the largest specialized unit for burns and plastic surgery in Uganda. These investigations are key to improving patient care directly and contribute to closing the gap on missing AMR surveillance data from a high-burden setting.

## 2. Materials and Methods

Patients of all age groups admitted to the specialized unit for burns and plastic surgery of Kiruddu National Referral Hospital (NRH) in Kampala, Uganda, with clinical signs of wound infections and/or fever were eligible for enrollment in this prospective observational cohort study. Clinical signs of wound infection included pus, conversion from partial- to a full-thickness wound, rapidly extending cellulitis surrounding the burn injury, eschar separation and tissue necrosis.

### 2.1. Study Setting

The burns and plastic surgery unit of Kiruddu NRH is the largest public specialized burn unit in Uganda. The unit has a capacity for 60 inpatients along with two operating theatres and a daily outpatient clinic with an estimated 1000 visitations annually. In addition, the unit has 10 intensive care beds for patients requiring non-invasive monitoring and oxygen supply. Wound debridement, scar correction and skin grafting are performed in the operating theatres connected to the unit. Generally, up to two thirds of the patients are children under the age of five years. Common reasons for burns include accidents involving hot liquids, charcoal and open fire cooking, electric burns, road traffic accidents and, less frequently, criminally motivated burns involving chemicals, notably battery acid.

All burn wounds are dressed with silver sulphadiazine cream of 1% and saline moistened, as well as povidone soaked gauze, with paraffin gauze as the non-adherent first layer. Surgical treatment includes debridement, escharotomy and fasciotomy for circumferential finger, hand, limb or torso burns, escharectomy to excise dead skin and avoid the compartment syndrome and skin grafting to cover clean deep burn wounds. Empirical antibiotic treatment for wound and/or systemic infections is chosen in line with national guidelines [20].

### 2.2. Study Procedures

Upon giving written informed consent, patients were consecutively screened for eligibility and enrolled by trained study staff. Information on demographics, past and present medical history, details of the reason for admission and history of antibiotic treatment were obtained. Wound infections were characterized as nosocomial in accordance with the WHO definition for healthcare-associated infections [21]. Blood samples were collected for general laboratory tests, the exclusion of acute malaria and for two pairs of blood cultures. Wound swabs were taken from all sites with clinical signs of infection. Blood cultures (BDBACTEC™, Becton/Dickinson, Franklin Lakes, NJ, USA) and wound swab samples were sent to the Department of Microbiology at Makerere University (accredited by the College of American Pathologists) for the immediate analysis of bacterial growth and antibiotic susceptibility testing (AST). The Kirby–Bauer disk diffusion method was used to determine the antimicrobial susceptibility based on the break points that correlate zones of inhibition with the minimum inhibitory concentrations of known antimicrobial agents [22]. Multidrug resistance (MDR) was defined as an acquired non-susceptibility to at least one agent in three or more antimicrobial categories [23]. Further laboratory procedures and microbiological diagnostics have been described in detail elsewhere [24]. 

Follow-up study visits conducted on day 2 and 4 included a focused physical examination, the documentation of current antibiotics and surgical treatments and an assessment of the treatment progress. Upon discharge from the unit, the final study visit included an outcome assessment, which was defined as follows: complete recovery, minor disability (patient can perform all activities of their daily life with minimal disturbance), moderate disability (patient is unable to perform some activities of their daily life without help and/or has a loss of digits excluding the forefinger, thumb or big toe) or major disability (patient is unable to perform most activities of their daily life without help and/or experienced the loss of vital digits such as the thumb, forefinger, big toe, or the loss of whole limbs or appendages such as eyes or ears). 

### 2.3. Data Management and Analysis

Data were collected and managed using the web-based software platform REDCap (Research Electronic Data Capture) hosted at the Infectious Diseases Institute, College of Health Sciences, Makerere University [25,26]. The data were exported into Microsoft Excel 2016 (Microsoft Corporation, Redmond, WA, USA) and STATA version 16.1 (StataCorp, College Station, TX, USA) for descriptive analysis by frequencies, percentages, median and interquartile ranges. Susceptibility and resistance rates to antibiotics were calculated for each recorded pathogen in percentages.

## 3. Results

### 3.1. Study Population

Between October 2020 and April 2022, 140 patients were included in this study, of which 62 (44.3%) were female. The median age at enrollment was 26 (IQR 7–35) years. The overall age distribution was widespread with two peaks in the age groups below 4 years (*n* = 31; 22.1%) and between 25 and 34 years (*n* = 39; 27.9%). The majority (*n* = 119, 85%) had no history of chronic illness. Five participants were HIV-positive, all of which were on antiretroviral treatment (ART). Most study participants primarily suffered from burn injuries (*n* = 101; 72.2%), which were classified according to the degree. Additional information on the affected Total Body Surface Area (TBSA) was not collected. The remaining study participants were admitted due to other trauma (*n* = 39; 27.8%) requiring plastic surgery and/or skin grafting. Table 1 shows the demographics and baseline characteristics of the study population.

The main reason for the study inclusion was wound infection (*n* = 105; 75%). Three patients (2.1%) were enrolled due to a fever and 32 (22.9%) patients were presented with both a fever and clinical signs of a wound infection. A total of 51 (36.4%) participants had a blood pressure (BP) > 100 bpm, 7 (5%) a systolic BP < 100 mmHg and 3 (2.1%) were both tachycardic and hypotonic. Infections were classified as nosocomial in 72 (51.4%) study participants.

**Figure 1:**
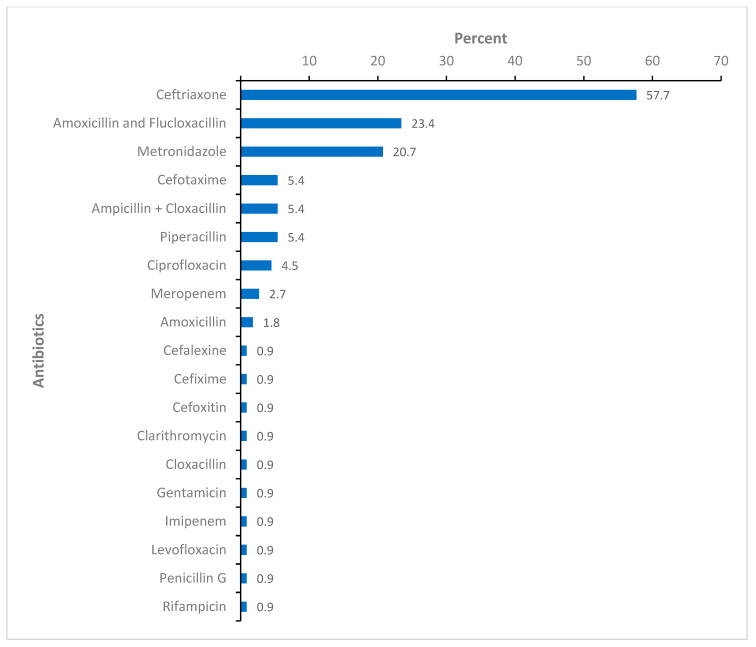
The antibiotic treatment at enrollment, *n* = 111. Figure legend: The percentage computed out of patients on antibiotic treatment at enrollment.

Concerning antibiotic treatment history, 47 (33.6%) participants reported having taken antibiotics for any reason at least once during the past year, most commonly ampicillin plus cloxacillin (*n* = 39, 83%), mostly purchased at drug shops or pharmacies (*n* = 29, 63%). Upon admission to the unit, the majority of the study participants (*n* = 111, 79.3%) were already on antibiotic treatment due to their burn injuries following transfer from health centers or other public hospitals. The most common empirical antibiotic treatment given was ceftriaxone (*n* = 64/111, 57.7%), followed by amoxicillin plus flucloxacillin (*n* = 26/111, 23.4%). Figure 1 shows the empirical antibiotic treatments at enrollment.

### 3.2. Microbiological Findings

**Figure 2:**
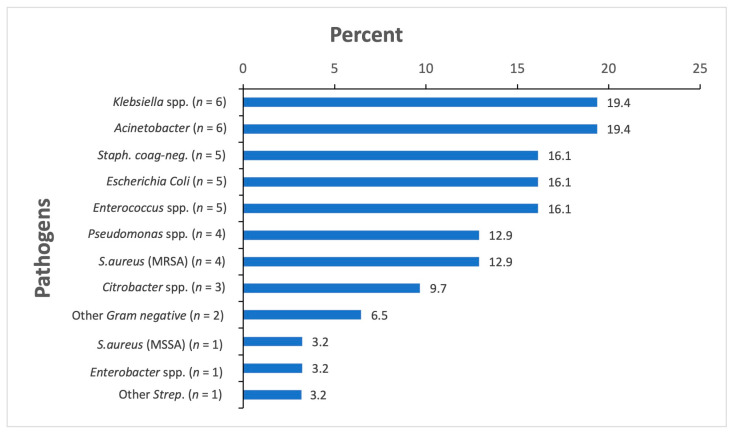
The bacteria isolated in blood cultures, *n* = 31. Figure legend: *n* denotes the number of patients with a positive blood culture for bacterial pathogens.

The blood culture results revealed bacteremia in 31 (22.1%) participants. Figure 2 shows the identified pathogens. All three *E.coli* isolates were resistant to ceftriaxone/cefotaxime, as well as 50% (3/6) of *Klebsiella* spp. isolates. For *Acinetobacter* spp., all isolates (6/6) were resistant to piperacillin/tazobactam, ciprofloxacin and gentamicin, while 40% (2/5) were resistant to amikacin and 33.3% (2/6) to carbapenems. Only one study participant was found to have Methicillin-resistant *Staphylococcus (S.) aureus* (MRSA) in a blood culture.

**Figure 3:**
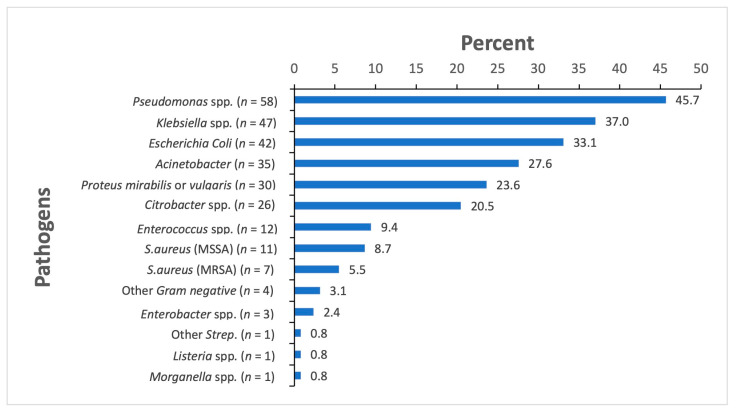
The bacteria isolated in wound swabs, *n* = 127. Figure legend: *n* denotes the number of patients with a bacterial pathogen isolated from wound swabs.

The wound swab culture results showed bacterial growth in 127 of 134 (94.8%) study participants with clinical wound infections. A total of 270 pathogens were identified and underwent AST. The most commonly identified pathogens from wound swabs were gram-negative bacteria including *Pseudomonas* spp. (45.7%), *Klebsiella* spp. (37.0%), *E. coli* (33.1%), *Acinetobacter* spp. (27.6%) and *Proteus (P.) mirabilis* or *P. vulgaris* (23.6%) (Figure 3).

Among the *Pseudomonas* spp. isolates, 74.4% (29/39) showed resistance to the fourth generation cephalosporine cefepime. Furthermore, *Pseudomonas* spp. isolates were resistant to ceftazidime, amikacin and gentamicin in 69.2% (27/39), 65% (26/40) and 65.9% (29/44) of cases, respectively. For *Klebsiella* spp., resistance to ceftriaxone/cefotaxime was seen in 95.6% (44/46) of the isolates, while resistance to amoxicillin/clavulanic acid and imipenem were found in 51.7% (15/29) and 8.5% (4/47) of isolates, respectively. *E. coli* isolates were resistant to ceftriaxone/cefotaxime in 87.5% (35/40), whereas carbapenem resistance was rarely observed (2 isolates). Almost half of all *E. coli* isolates were resistant to amoxicillin/clavulanic acid (45.5%,15/33) and more than half were resistant to ciprofloxacin (52.8%, 19/36). *Acinetobacter* spp. were isolated in 27.6% (35/127) of positive wound swab cultures. Here, 45.5% (15/33) of isolates were resistant to carbapenems, 67.9% (19/28) were resistant to piperacillin/tazobactam and 77.4% (24/31) were resistant to fluoroquinolones. Among *P. mirabilis* or *P. vulgaris* isolates, 14.3% (4/28) were resistant to carbapenems, 6.3% (1/16) were resistant to piperacillin/tazobactam, and 23.5% (4/17) showed resistance to amoxicillin/clavulanic acid. 

Extended-spectrum beta-lactamase (ESBL) production was recorded in 71.0% (66/93) of gram-negative pathogens identified in wound swabs. The most common ESBL-producing pathogens were the *Klebsiella* spp. With 76.6% (36/47 of isolates), followed by *E. coli* with 66.7% (28/42). None of the wound swab culture isolates revealed a growth of MRSA. Table 2 shows resistance patterns of the six most commonly identified pathogens.

### 3.3. Targeted Antibiotic Treatment

For the majority of study participants (*n* = 89, 63.6%), the identified pathogens were resistant to the initial choice of the empirical antibiotic treatment according to the AST results. Only approximately one fifth (*n* = 29, 20.7%) of the participants had received an empirical treatment to which the identified pathogens were fully susceptible. The remaining participants (*n* = 22, 30.8%) were either not on empirical treatment or no pathogen was identified. Following AST, 58 (41.4%) patients received a switch to targeted antibiotic treatment, most commonly with fluoroquinolones (25.3%) or carbapenems (19.3%).

### 3.4. Surgical Treatment and Clinical Outcome

The median stay at the ward was 46 days (IQR 29–66). Most study participants (*n* = 91, 65%) received split-skin grafts, while a smaller proportion (*n* = 7, 5%) were treated only with surgical wound debridement or other surgical treatment (*n* = 14, 10%). In two cases (1.4%) amputation of at least one extremity was performed. At the end of the follow-up period, 21 (15%) study participants showed a favorable clinical outcome with complete recovery. Forty-one (29.3%) study participants remained with minor disabilities, 52 (37.1%) with moderate disabilities and 14 (10%) with major disabilities. Twelve (8.6%) study participants died. Most patients (*n* = 8) died of sepsis due to multidrug resistant pathogens, predominantly *E.coli*, *Klebsiella* spp. and *Acinetobacter baumannii*. Further causes of death included a pulmonary embolism (*n* = 1), aspiration pneumonia (*n* = 1), falciparum malaria (*n* = 1) and malnutrition with severe burns (*n* = 1).

## 4. Discussion

We report here the clinical and microbiological findings of patients with wound infections admitted to the specialized burns unit of Kiruddu NRH in Kampala, Uganda. Most patients had suffered grade 2b or 3 burn wounds due to flames and were referred from other health facilities for specialized surgical treatment, primarily skin grafting. Chronic illness was rare in our population. The majority of study participants (79.3%) were on empirical antibiotic treatment at enrollment. Due to incomplete documentation from the transferring health centers and hospitals, we were unable to report the type and duration of the antibiotic treatment prior to enrollment. While blood cultures were rarely positive in our study population, the microbiological analysis of swabs from clinically infected wounds revealed multidrug resistant gram-negative pathogens in all cases with very limited treatment options. ESBL production was identified in 71.0% of pertinent gram-negative isolates. While Ugandan National Guidelines recommend a combination of benzylpenicillin +/− gentamicin in case of systemic signs of infection in patients suffering from burn wounds, the most common empirical antibiotic treatment prescribed in our cohort was ceftriaxone. Ceftriaxone is preferred because clinicians do not expect sensitivity to penicillin, and gentamycin is avoided due to its toxicity and the common occurrence of acute kidney injury due to the burn injury itself. The empirical treatment with ceftriaxone was commonly chosen, as it is easily accessible, available free of charge, considered highly potent and well tolerated. However, the identified pathogens within our study cohort were resistant to this choice of treatment in 95.6% of *Klebsiella* spp. isolates, 87.5% of *E. coli* isolates and 70.8% of *Citrobacter* spp. isolates, making this treatment option inappropriate for our study population. 

Our findings highlight the need for routine access to microbiological diagnostics. Although severe wound infections with resistant pathogens are a common complication in patients with burns globally, specific data from Ugandan patients and other sub-Saharan African regions are scarce [27]. Treatment guidelines for empirical antibiotic therapy of such patients are not necessarily based on the expected pathogens and common resistance profiles, but on drug availability and the hope of preserved susceptibility [12]. To directly improve patient care and contribute to local surveillance data, accurate microbiological diagnostics are key, especially in patients with burn wounds prone to infections with resistant pathogens [27]. Similar to the development of generic drug options for the treatment of infectious diseases in low-resource settings, microbiological diagnostics need to become accessible.

A large proportion of infections within our study population were classified as nosocomial, which is further supported by our microbiological findings. In this context, it is worth mentioning that there is no specific hygiene concept in place in the unit—including patient isolation upon the detection of multidrug resistant pathogens. While all surgical materials are sterilized professionally in-house, patient surroundings, showers and dressing rooms can be a source of infection, which warrants further exploration and improved infection prevention practices.

In this setting, the over-the-counter sale of antibiotics is a further driver of AMR [28]. A large proportion (33.6%) of our study participants reported having received antibiotics at least once during the previous year, most of which were purchased in drug shops or pharmacies (63%). Important oral treatment options such as ciprofloxacin and doxycycline are commonly sold [29]. To preserve these substances, over-the-counter availability needs to be critically evaluated. Systems to secure the rational use of antibiotics and other anti-infective treatments are highly warranted [30].

Our study setting is not entirely comparable to regular patient care, as the treatment was based on microbiological results and expensive drug options were made available for participants with infections due to resistant pathogens. However, despite the study-related improved care, the overall mortality among study participants was high (8.6%). The main cause of death was gram-negative sepsis, predominantly due to multidrug resistant strains of *E. coli*, *Pseudomonas* spp. and *Acinetobacter* spp. The timely availability of the targeted combination of antimicrobial treatments including reserve antibiotics such as colistin might save lives in this context [31].

While our patient sample size is sufficient to report clinical findings in this special study population, our microbiological findings are partly limited by the small number of isolates for the selected pathogens. However, in the context of the scarce availability of local AMR surveillance data, we consider it highly important to report even small numbers. In-line with the WHO Global Action Plan on AMR, our findings aim to contribute to increasing awareness through surveillance and research from an underrepresented region of the world [32]. 

## 5. Conclusions

Our study population was highly affected by resistant, predominantly gram-negative pathogens with very limited antibiotic treatment options for wound infections. First-choice empirical treatment with ceftriaxone seems to not be an option in the context of infected burn wounds. In order to improve the clinical outcomes of patients with burn injuries and other reasons for surgical reconstruction in this setting, the prevention of nosocomial infections, routine access to microbiological diagnostics and the timely availability of targeted antibiotic treatments are necessary.

## Figures and Tables

**Table 1:** The demographics and baseline characteristics, *n* = 140.

Demographics	
Sex, *n* (%)	
Male	78 (55.7)
Female	62 (44.3)
Age in years, median (IQR)	26 (7–35)
Length of hospital stay at enrollment in days, median (IQR)	8.5 (4–15.5)
Length of hospital stay at termination in days, median (IQR)	45.5 (28.5–66.0)
Reason for admission, *n* (%)	
Plastic reconstruction after burn injury	101 (72.2)
Orthopedic/Trauma	36 (25.7)
Post-operative	3 (2.1)
Type of Orthopedic/Trauma case ^1^	
Trauma without fractures	15 (41.7)
Fracture of lower limbs	9 (25.0)
Other trauma	7 (19.4)
Polytrauma	5 (13.9)
Causes of burns ^2^	
Flames	42 (41.6)
Hot other liquids	23 (22.8)
Hot water	15 (14.9)
Electricity	15 (4.9)
Acid	3 (3.0)
Others	7 (6.9)
Degree of burn at enrollment, *n* (%) ^3^	
2b (deep partial thickness)	73 (72.3)
3 (full thickness without bone involvement)	15 (14.9)
2a (superficial partial thickness)	8 (7.9)
4 (full thickness with bone involved)	5 (4.9)

Table legend: IQR = inter-quartile range. ^1^ Denominator is the number of patients with orthopedic/trauma as the reason for admission (*n* = 36). ^2^ Multiple causes of burns in one patient possible. ^3^ Denominator is the number of patients admitted due to a burn injury (*n* = 101).

**Table 2:** The resistance to antimicrobials for six most common bacterial pathogens ^1^.

Isolates	*Pseudomonas* spp. N = 56 *n*/N (%)	*Klebsiella* spp. N = 47 *n*/N (%)	*E. coli* N = 42 *n*/N (%)	*Acinetobacter* spp. N = 35 *n*/N (%)	*Proteus* spp. N = 30 *n*/N (%)	*Citrobacter* spp. N = 26 *n*/N (%)
Amoxicillin/Clavulanic Acid	-	15/29 (51.7)	15/33 (45.5)	-	4/17 (23.5)	9/14 (64.3)
Ampicillin	-	32/32 (100)	21/21 (100)	-	14/17(82.3)	16/16 (100)
Piperacillin	30/48 (62.5)	5/5 (100)	4/4 (100.0)	26/31(83.9)	4/4 (100)	2/2
Piperacillin/Tazobactam	15/41 (36.6)	7/27 (25.9)	9/19 (47.4)	19/28 (67.9)	1/16 (6.3)	4/16 (25.0)
Cefuroxime	-	42/44 (95.5)	29/33 (87.9)	-	18/25 (72.0)	20/23 (86.9)
Cefotaxime OR Ceftriaxone	1/1	44/46 (95.6)	35/40 (87.5)	8/11 (72.7)	16/29 (55.2)	17/24 (70.8)
Ceftazidime	27/39 (69.2)	34/39 (87.2)	23/25 (92.0)	23/28 (82.1)	8/14 (57.1)	12/20 (60.0)
Cefepime	29/39 (74.4)	2/2 (100.0	9/9 (100)	21/27 (77.8)	-	3/3 (100)
Imipenem OR Meropenem	2/53 (3.8)	4/47 (8.5)	2/39 (5.1)	15/33 (45.5)	4/28 (14.3)	6/26 (23.1)
Ciprofloxacin	11/48 (22.9)	14/45 (31.1)	19/36 (52.8)	24/31 (77.4)	16/28 (57.1)	8/26 (30.8)
Amikacin	26/40 (65.0)	1/20 (5.0)	2/21 (9.5)	8/25(32.0)	1/13 (7.7)	2/13 (15.4)
Cotrimoxazole	2/2	28/28 (100)	23/27 (85.2)	10/10 (100)	13/14 (92.9)	15/18 (83.3)
Gentamicin	29/44 (65.9)	22/37 (59.5)	13/30 (43.3)	24/30 (80.0)	12/25 (48.0)	12/18 (66.7)
Chloramphenicol	1/1	20/33 (60.6)	10/30 (33.3)	-	15/22 (68.2)	8/12 (66.7)
Colistin/Polymyxin B	-	-	-	-	-	1/4 (25.0)
Tetracycline	-	-	-	18/27 (66.7)	-	-

Table legend: ^1^ Pathogens identified in wound swabs.

## Data Availability

The data presented in this study are available on request from the corresponding author.
